# SURVEILLANCE AND MONITORING: CDC Releases Updated Biomonitoring Report

**DOI:** 10.1289/118-a66a

**Published:** 2010-02

**Authors:** Bob Weinhold

**Affiliations:** **Bob Weinhold**, MA, has covered environmental health issues for numerous outlets since 1996. He is a member of the Society of Environmental Journalists

Biomonitoring—the science of measuring environmental chemicals in human blood, urine, and other tissues—made another modest advance with the 10 December 2009 release of the *Fourth National Report on Human Exposure to Environmental Chemicals* by the Centers for Disease Control and Prevention (CDC). The report includes data on 75 substances not in the preceding report, for a total of 212 reported chemicals. Data for more than 45 additional pesticides and metabolites are expected to begin appearing on the CDC website (http://www.cdc.gov/exposurereport/) by spring 2010.

This is almost a 10-fold increase over the 27 substances covered in the initial report in 2001, greatly expanding the data researchers can use in their continuing efforts to take the next step—determining what the reported concentrations mean from a health risk perspective. “Interpretation is key to making biomonitoring data more meaningful,” says Sarah Brozena, senior director for regulatory and technical affairs with the American Chemistry Council. Yet such understanding is extremely limited so far for most chemicals.

But the science of biomonitoring may be nearing a short-term zenith for the number of substances assessed in the general population, and future CDC reports “will probably grow less than [they] have recently,” says John Osterloh, chief medical officer and toxicologist with the CDC Division of Laboratory Sciences. That’s due to technological limitations as well as capacity and budget constraints. As a result, in the foreseeable future it’s likely that data for only a tiny fraction of the 239,000 toxic substances listed by the Chemical Abstract Service as regulated or included in inventories worldwide would be included in biomonitoring efforts.

The report draws on data from the National Health and Nutrition Examination Survey, an ongoing survey that every 2 years samples a small number of people intended to represent the U.S. general population. Chemicals included in the fourth report have been selected on the basis of likelihood of exposure in the U.S. population, seriousness of known or suspected health effects resulting from exposure, and availability of appropriate ways to measure the chemical, among other factors. Of the samples assessed, 90–100% had detectable levels of substances such as perchlorate, mercury, bisphenol A, acrylamide, multiple perfluorinated chemicals, and the flame retardant BDE-47.

Osterloh knows of no breakthrough technologies that can substantially expand the CDC biomonitoring program. One option could be to have industries generating chemicals conduct such testing—a suggestion that could be presented in future legislative proposals or efforts to revise the Toxic Substances Control Act. In the meantime, biomonitoring programs in state public health laboratories, Canada, and Europe offer no near-term prospects for covering substantially more substances.

Another important limitation of the CDC’s current effort is that it covers just one substance at a time. “In the real world, we’re exposed to mixtures,” says Anila Jacob, a senior scientist with the Environmental Working Group, an advocacy organization that has documented 414 substances in 186 people it has tested, many of whom carry scores of contaminants. While Osterloh acknowledges concerns about the combined effects of chemicals, he says the CDC’s current program isn’t designed to address mixtures. However, he adds the CDC plans to evaluate mixtures for some chemicals, such as the dioxin-like chemicals, in separate scientific endeavors.

The current CDC biomonitoring methodology is not designed to assess geographic differences in exposure, nor can it be used to assess fetal contamination. The latter is a particular concern for Jacob, whose team found up to 232 contaminants in a recent study of cord blood from 10 newborns. “The developing fetus is one of the most vulnerable populations out there,” she says. However, a pilot study of cord blood from infants born to 525 women that is on tap as part of the blossoming National Children’s Study (of which CDC is a part) could begin to help remedy this shortcoming.

